# Relationship Between Physical Activity and Function With Quality of Life in Community-Living Older Adults

**DOI:** 10.1093/geroni/igaa057.613

**Published:** 2020-12-16

**Authors:** Milan Chang, Olof Geirsdottir, Inga Thorsdottir, Palmi Jonsson, Alfons Ramel, Milan Chang Gudjonsson

**Affiliations:** 1 University of Iceland, Reykjavik, Iceland; 2 University of Iceland, Reykjavik, Capital area, Iceland

## Abstract

Background: Quality of life (QOL) is a multidimensional concept which is often used as an evaluation of a person‘s health and psychological status. Increasing longevity can be associated with better QOL as long as older adults are independent in daily life. The aim of the study was to examine the associations of QOL with muscle strength and physical function among community-dwelling older adults. Methods: The current cross-sectional study had 225 participants (73.7±5.7yrs, 58.2% female) living in Reykjavik, Iceland. QOL measured using the 36-item short-form survey (SF-36). Covariates were anthropometrics, muscle strength, physical function including timed up and go test (TUG), and 6-minute walking distance (6MWD), physical activity per week (PA). Linear regression analysis was used to examine the association of QOL with physical function. Results: The mean QOL score for the study population was 54.9±6.13. The analysis was adjusted for age and gender, body mass index, height, and PA. We found that QOL was associated with better grip strength (B=1.4, P<0.0001), 6MWD (B=0.03, P<0.0001), slower TUG (B=-0.9, P<0.0001), and higher PA (B=0.03 m, P=0.039). However, QOL was not associated with quadriceps leg strength. Conclusion: The study suggests that QOL was associated with better physical function including grip strength, walking ability and the level of PA among community-dwelling older adults in Iceland.

